# Dietary Effects of Fermented Cottonseed Meal Substituting Fishmeal on the Growth, Biochemical Indexes, Antioxidant Capacity, and Muscle Quality of Juvenile Golden Pompano (*Trachinotus ovatus*)

**DOI:** 10.1155/2024/9972395

**Published:** 2024-06-19

**Authors:** Zhanzhan Wang, Shuling Liao, Zhong Huang, Jun Wang, Yun Wang, Wei Yu, Xiaolin Huang, Maoyan Luo, Heizhao Lin, Chuanpeng Zhou

**Affiliations:** ^1^ Key Laboratory of Aquatic Product Processing Ministry of Agriculture and Rural Affairs South China Sea Fisheries Research Institute Chinese Academy of Fishery Sciences, Guangzhou 510300, China; ^2^ School of Fisheries Tianjin Agricultural University, Tianjin 300384, China; ^3^ School of Life Science Guangzhou University, Guangzhou 510006, China; ^4^ Shenzhen Base of South China Sea Fisheries Research Institute Chinese Academy of Fishery Sciences, Shenzhen 518121, China; ^5^ Key Laboratory of Efficient Utilization and Processing of Marine Fishery Resources of Hainan Province Sanya Tropical Fisheries Research Institute, Sanya 572018, China

## Abstract

This study investigated the effects of the dietary replacing fishmeal (FM) with fermented cottonseed meal (FCSM) on growth performance, body coloration, serum biochemistry, muscle quality, and liver antioxidant capacity of juvenile golden pompano (*Trachinotus ovatus*). Fish were fed with five experimental diets (0 (FM), 12.5% (CSM12.5), 25% (CSM25), 50% (CSM50), and 100% (CSM100) replacement levels) for 8 weeks. The weight gain rate (WGR), specific growth rate (SGR), and condition factor (CF) in fish fed with CSM25 were significantly higher than those of the FM (*P* < 0.05). ALT, GLU, TG, TC, and LDL of fish fed with CSM100 diet were significantly higher than those in FM (*P* < 0.05). No significant difference was observed in SOD, CAT, and MDA among all treatments (*P* > 0.05). The relative gene expression of *Nrf2* of fish fed with CSM25 diet was higher than that of the other groups (*P* < 0.05). The relative gene expression of *Keap-1* of fish fed with CSM25 diet was lower than those in FM (*P* < 0.05). In addition, the replacement of a high proportion of FM with FCSM negatively affect the liver antioxidant capacity of fish. With dietary replacement of FM with FCSM increasing 0%–25%, the relative expressions of *GH*, *myf5*, and *MSTN* were significantly upregulated (*P* > 0.05). Based on these results, we recommend that of FCSM in the diet of golden pompano, whereas the optimal level of FCSM should be carefully evaluated. In conclusion, the optimum level of dietary replacing FM with FCSM in *T. ovatus* diet was 24.74%−29.38% based on SGR and WGR.

## 1. Introduction

Fishmeal has many advantages, such as rich amino acid composition, high protein content, low carbohydrate content, low antinutritional factors, and good palatability [1, 2]. In recent years, the availability of fishing sites worldwide has decreased significantly due to indiscriminate fishing and other human activities [3]. As a result, there has been a steady decline in global fishmeal production [4]. Conversely, the rapid growth of the aquaculture sector has led to an oversupply of fishmeal, resulting in a steady increase in its price, thus driving up the cost of aquatic feed [5]. Currently, many researchers have tried to substitute fishmeal in diets with high yield, cheap, high utilization, and environmental friendliness [1] animal protein [6], plant protein [7], and single-cell proteins [8].

There are several plant protein sources that are being studied as potential alternatives to fishmeal in aquatic animal nutrition and feed research. These sources include fermented soybean meal protein, soybean protein concentrate, peanut cake meal protein, cottonseed meal protein, and corn meal protein [7]. The question of whether the replacement of fishmeal with plant protein sources has any adverse effects on aquatic animals has also been answered differently by many researchers [2]. In marine animals such as red-tailed catfish (*Hemibagrus wyckioides*) [9], and red snapper (*Pagrus major*) [10], the substitution of 11.2% and 56% of fishmeal by rapeseed meal, respectively, was found to have no effect on the growth performance of aquatic animals. On the other hand, studies on the substitution of fishmeal by peanut meal found that in hybrid grouper (*Epinephelus fuscoguttatus♀* × *Epinephelus lanceolatus♂*), when the substitution ratio reached 50%, although it did not significantly reduce their growth performance, it significantly altered their immune capacity and intestinal flora structure [11]. Researchers have also investigated the reasons for the negative effects of substituting fishmeal with plant protein sources on the growth of aquatic animals [7]. First, animal proteins are superior to plant proteins in terms of palatability [12]. Second, the digestibility of plant protein is inferior to that of animal protein [13]. Third, plant protein contains antinutritional factors that inhibit the feeding and growth of aquatic animals, etc. [14]. Antinutritional components found in plant protein sources can have varying degrees of detrimental effects on aquatic animals [15]. These effects include reduced feed utilization, tissue or organ damage, and impaired nutrient absorption from the diet [16].

China produces an enormous amount of cottonseed meal. Compared with fishmeal, cottonseed meal has a lower dependence on foreign imports, resulting in a lower price [17]. In addition, cottonseed meal has a balanced and higher protein content, making it more nutritionally valuable [18]. Therefore, the substitution of fishmeal with cottonseed meal in aquatic feeds is crucial for the development of our aquaculture sector. Research carried out on *Sillago sihama Forskál* showed that replacing 16% of the fishmeal with low gossypol cottonseed meal had no adverse effects on the fish [19]. The major reasons for limitations utilization of cottonseed meal in aquafeed were probably due to the two reasons. First, it includes antinutritional components, specifically free cottonseed phenol, which can be detrimental to fish if taken excessively [20]. It can also bind to lysine and iron, reducing their effectiveness [21]. Second, cottonseed meal has a low content of lysine and other essential amino acids, and the digestibility of essential amino acids is significantly lower than that of fishmeal [15].

*Trachinotus ovatus* is a carnivorous fish with many characteristics, such as fast growth, high flesh quality, and easy domestication. The experiment was undertaken to assess the impacts of replacement of fishmeal (FM) with fermented cottonseed meal (FCSM) for golden pompano on the basis of the growth performance, body coloration, serum biochemistry, muscle quality, and liver antioxidant capacity.

## 2. Materials and Methods

### 2.1. Ethical Statement

The experimental protocol followed that of the Institutional Animal Care and Ethics Committee of the South China Sea Fisheries Research Institute, Chinese Academy of Fishery Sciences, Guangzhou, China.

### 2.2. Diet Preparation

Five isonitrogenous (~42% crude protein) and isolipidic (~11.8% crude lipid) experimental diets were formulated with different levels of replacing FM with FCSM of 0 (FM), 12.5% (CSM12.5), 25% (CSM25), 50% (CSM50), and 100% (CSM100), respectively. The formulation and ingredient composition of the experimental diets are shown in Table 1. Fishmeal, casein, soybean protein concentrate, and soybean meal were used as protein. Fish oil and soybean lecithin were used as lipid sources. The cottonseed meal was dried and then ground into powder. FCSM powder (10 kg) was prepared by solid fermentation with complex probiotics of *Lactobacillus plantarum* (3.7 × 10^9^ CFU/g), and *Bacillus subtilis* (7.7 × 10^8^ CFU/g) in the laboratory. In total, 100 g of FCSM powder was mixed with 0.5 g of complex probiotics which was dissolved in 29 mL of sterile water. After being fermented at 35°C for 5 days. The fermentation bacteria are all from Jiangsu Su Wei Institute of Microbiology Co., Ltd., (WuXi 214063, China). To eliminate limiting amino acids [22], lysine and methionine were supplied to each group to equalize their amino acid content.  Table 2 shows the amino acid composition of the experimental diets. All the ingredients were ground into powder, sieved through 60 mesh, and thoroughly mixed with oil and water. The 2.5 and 3.0 mm diameter long doughs were extruded using a twin-screw extruder (F-26, South China University of Technology, Guangzhou, China), cut into pelleted diets using a pelletizer (G-500, South China University of Technology, Guangzhou, China), and then stored at −20°C in the refrigerator until use.

### 2.3. Fish Rearing and Experimental Conditions

The feeding trial was conducted in a seawater pond at the Shenzhen Base of the South China Sea Fisheries Research Institute of the Chinese Academy of Fishery Sciences (Shenzhen, China). Juvenile golden pompano were acclimated to the experimental system and fed with the commercial diet (crude protein ≥ 43.0%, crude fat ≥ 8.0%, Guangdong Yuequn Biotechnology Co., Ltd., Guangzhou, China) for 2 weeks. Before feeding trial, fish were starved for 24 hr. Subsequently, a total of 375 fish with a mean initial body weight of 5.6 ± 0.14 g were selected and randomly assigned into 15 net cages. The net cages were randomly divided into five groups with three replicates per group. Fish were artificially fed twice daily at 6 : 00, and 18 : 00 until apparent satiety. During the feeding trial, the temperature was maintained at 28.3–33.3°C. Dissolved oxygen was higher than 6.0 mg/L. Salinity and ammonia were in the range of 20–25 g/L and 0.05–0.1 mg/L, respectively. The photoperiod was the natural day–light cycle throughout the experimental period.

### 2.4. Sample Collection

Fish were anesthetized with 0.01% overdose eugenol (Shanghai Reagent Corp., China), and then fish in each net cage were counted and weighed. Five fish from each net cage were randomly selected and measured in weight and body length to calculate morphology index. Three fish from each cage were sampled and stored at −20°C for whole-body composition analysis. Blood samples were collected from the caudal vein of 10 fish per cage. After centrifugation at 4°C (3,000x *g*, 10 min), the plasma was collected for determining serum biochemical parameters. Three fish were collected from each cage and the whiteness (*L* ^*∗*^), redness (*a* ^*∗*^), yellowness (*b* ^*∗*^), and total color difference (*ΔE* ^*∗*^) of the abdomen and back of the fish were measured using a colorimeter (GEB-104 Pantone Color-Cue). The dorsal measurement area refers to the region located between the lower part of the leading edge of the dorsal fin and the upper part of the lateral line. The ventral measurement area is the area between the ventral fin and the anal fin of the ventral side of the golden pompano. Prior to measurement, the water on the surface of the fish was sucked dry with absorbent paper. Part of the liver samples from three fish in each cage were immediately frozen in liquid nitrogen and then stored in a −80°C refrigerator for subsequent determination of enzyme activity. Liver and muscle from three fish in each cage were frozen in liquid nitrogen and then stored at 80°C until extracted total RNA.

### 2.5. Parameters Measurement and Analysis

#### 2.5.1. Proximate Composition Analysis

The chemical analysis of the diets and ingredients was determined according to the standard methods of the Association of Official Analytical Chemists [23]. The samples were dried in an oven at 105°C for 72 hr to determine moisture. Crude protein (*N* × 6.25) was determined with an automated Kjeldahl analyzer (Kjeltec 8400, FOSS, Hoganos, Sweden). Lipid levels were quantified by ether extraction using a Soxhlet apparatus (Soxtec 2055, FOSS, Hoganos, Sweden). Ash was determined using a muffle furnace (FO610C, Yamato Scientific Co., Ltd., Japan) at 550°C for 5 hr. Amino acids in the diets were determined after hydrolysis using an amino acid analyzer (Hitachi, Tokyo, Japan).

#### 2.5.2. Free Cotton Phenol Content Measurements

The free cotton phenol content analysis of the diets was determined according to the standard methods of the American Oil Chemists Society (AOCS 2009; method Ba 7b-96) [24]. All the samples were analyzed using an 8453 ultraviolet–visible spectrophotometer (Agilent Technology Co., Ltd., United States).

#### 2.5.3. Enzyme Activity Assay Analysis

Total protein (TP), glutamic oxaloacetic transaminase (ALT), alkaline phosphatase (ALP), glucose (GLU), triglycerides (TG), total cholesterol (TC), high-density lipoprotein cholesterol (HDL), low-density lipoprotein cholesterol (LDL), complement 3 (C3) and complement 4 (C4), superoxide dismutase (SOD), catalase (CAT), glutathione peroxidase (GSH-Px), total antioxidant capacity (T-AOC), and malondialdehyde (MDA) were determined using an automated analyzer (DR-200BS, Wuxi Huawei Delong Instrument Co., Ltd., China).

#### 2.5.4. Gene Expression Level Analysis

Total RNA of midintestine was extracted using Animal Total RNA Isolation Kit (FOREGENE Co., Ltd., Chengdu, China) according to the manufacturer's instructions. The quality of total RNA was tested by electrophoresis with 1% agarose gel, and the amount of total RNA was determined using a NanoDrop 2000 ultra-microspectrophotometer (Thermo Fisher Scientific, USA). The cDNA was synthesized from 1 *μ*g of total RNA using the Prime Script TM RT Reagent Kit with a gDNA Eraser Kit (Takara) according to the manufacturer's instructions. The cDNA was stored at 20°C for quantitative real-time PCR. The specific primers for the target genes and reference gene (*β*-actin) are shown in Table 3. All primer pairs required for quantitative real-time fluorescence PCR were synthesized by Sangon Biotech (Shanghai) Co., Ltd. Quantitative real-time PCR reaction was performed on the Light Cycler® 480 II Real-Time PCR System (Roche, Switzerland) using the S Dx PCR apparatus (ABI, USA). The reaction system of quantitative real-time PCR is as follows: 2× SYBR Green Pro Taq HS Premix 6.25 *μ*L, template cDNA 1 *μ*L, forward primer 0.5 *μ*L, reverse primer 0.5 *μ*L, RNase-free water 4.25 *μ*L. The qPCR protocol started with a predenaturation at 95°C for 30 s, followed by 40 cycles including denaturation at 95°C for 5 s and annealing and extension at 60°C for 30 s. Finally, a melting curve was stepped from 60 to 95°C gradually increasing 0.5°C/s. The relative expression of the target gene was calculated using the 2^−*ΔΔ*CT^ method [32].

### 2.6. Calculations

The parameters were calculated as per the following formulae:

(1) $$\begin{array}{c} \text{Survival rate }\left(\text{SR},\%\right)=100\times \left(\text{finial number of fish}/\text{initial number of fish}\right), \end{array}$$

(2) $$\begin{array}{c} \text{Weight gain rate }\left(\text{WGR},\%\right)=100\times \left(\text{(final body weight}-\text{initial body weight}\right)/\text{initial body weight)}, \end{array}$$

(3) $$\begin{array}{c} \text{Specific growth rate }\left(\text{SGR},\%\text{/day}\right)=100\times \left(\left(\text{ln finial individual weight}-\text{ln initial individual weight}\right)\right./\text{number of days)}, \end{array}$$

(4) $$\begin{array}{c} \text{Viscerosomatic index }\left(\text{VSI},\%\right)=100\times \left(\text{viscera weight (g)}/\text{whole body weight (g)}\right), \end{array}$$

(5) $$\begin{array}{c} \text{Hepatosomatic index }\left(\text{HSI},\%\right)=100\times \left(\text{liver weight (g)}/\text{whole body weight (g)}\right), \end{array}$$

(6) $$\begin{array}{c} \text{Condition factor }\left(\text{CF},g/{\text{cm}}^{3}\right)=100\times \left(\text{body weight }\left(g\right)/{\left(\text{body length (cm)}\right)}^{3}\right), \end{array}$$

(7) $$\begin{array}{c} \text{Feed intake }\left(\text{FI},\%\text{/day}\right)=100\times \left(\text{crude feed intake}/\text{ABW}/\text{day}\right),\left(\text{where ABW }\left(g\right)=\text{Average body weight}=\left(\text{Final body weight}+\text{initial body weight}\right)/2\right), \end{array}$$

(8) $$\begin{array}{c} \text{Feed conversion ratio}\left(\text{FCR}\right)=\text{Dry feed weight }\left(g\right)/\left(\text{Total final body weight}-\text{total initial body weight}\right), \end{array}$$

(9) $$\begin{array}{c} \text{Protein efficiency ratio}\left(\text{PER}\right)=\text{Fish weight gain }\left(g\right)/\text{Protein intake }\left(g\right). \end{array}$$

### 2.7. Statistical Analysis

Experimental data are presented as mean ± SEM. Statistical analysis was performed using SPSS 26.0 software (SPSS et al., USA) for Windows. All evaluated variables were subjected to an analysis of variance (ANOVA) to determine the significantly (*P* < 0.05) affected the observed responses. After passing the test, the experimental data were subjected to a one-way analysis of variance. If there were significant differences, the group means were further compared using Duncan's multiple-range test, and the probability *P* < 0.05 was considered significant.

## 3. Results

### 3.1. Growth Performance and Morphological Indexes of *T. ovatus*

**Figure 1:**
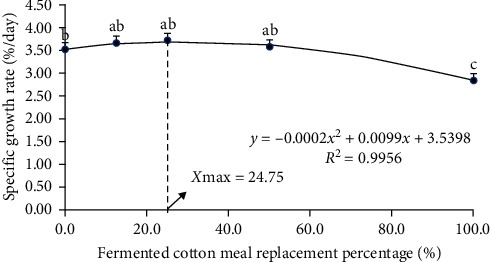
Relationship between the SGR and the level of replacement of fish meal by FCSM in the diets of *T. ovatus*. Means with different superscripts are significantly different (*P* < 0.05).

**Figure 2:**
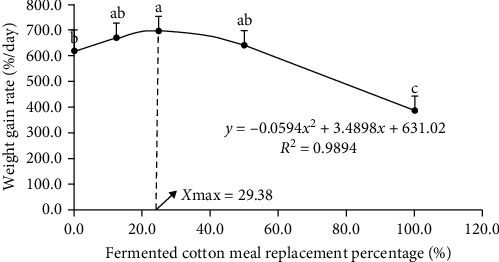
Relationship between the WG and the level of replacement of fish meal by FCSM in the diets of *T. ovatus*. Means with different superscripts are significantly different (*P* < 0.05).

As shown in Table 4, WGR and SGR of fish fed with CSM25 diet were significantly higher than those in FM (*P* < 0.05). SR, FI, and PER of fish fed with CSM100 diet were significantly lower than those in other groups, while the opposite was found for HSI and FCR (*P* < 0.05). The VSI of fish fed the CSM100 diet was significantly higher than those in FM, CSM12.5, and CSM25 (*P* < 0.05). CF of fish fed with CSM25 and CSM50 diets were significantly higher than those in FM and CSM12.5 (*P* < 0.05). The quadratic curve equation between the SGR of golden pompano and the proportion of replacement of FM with FCSM was *y* = −0.0002*x*^2^ + 0.0099*x* + 3.5398 (*R*^2^ = 0.9956) (Figure 1), from which the optimum proportion of replacement of FM with FCSM could be determined to be 24.75%. The quadratic curve equation between the WGR of golden pompano and the proportion of replacing FM with FCSM was *y* = −0.0594*x*^2^ + 3.4898*x* + 631.02 (*R*^2^ = 0.9894) (Figure 2), from which the optimum proportion of replacing FM with FCSM was determined to be 29.38%.

### 3.2. Body Coloration of *T. ovatus*

As shown in Table 5, the *a* ^*∗*^ of the dorsal skin of fish fed with the CSM100 diet was significantly higher than those in CSM12.5 (*P* < 0.05). No significant difference was observed in the *L* ^*∗*^ and *b* ^*∗*^ of dorsal skin, the *L* ^*∗*^, *a* ^*∗*^, and *b* ^*∗*^ of abdominal skin among all treatments (*P* > 0.05).

### 3.3. Determination of Biochemical Indexes of *T. ovatus*

The effects of replacing FM with FCSM on blood physiological indices are shown in Table 6. ALT, GLU, TG, TC, and LDL of fish fed with CSM100 diet were significantly higher than those in FM (*P* < 0.05). ALP of fish fed with CSM12.5 and CSM100 diets were significantly higher than those in FM (*P* < 0.05). HDL of fish fed the CSM50 diet was significantly lower than those in FM (*P* < 0.05). C3 of fish fed the CSM12.5 diet was significantly lower than those in FM (*P* < 0.05). C4 of fish fed the CSM100 diet was significantly lower than those in FM (*P* < 0.05).

### 3.4. Liver Antioxidant Capacity of *T. ovatus*

As shown in Table 7, GSH-Px of fish fed the CSM50 diet was significantly lower than those in FM (*P* < 0.05). T-AOC of fish fed with CSM25 diet was significantly higher than those in CSM50 (*P* < 0.05). No significant difference was observed in SOD, CAT, and MDA among all the treatments (*P* > 0.05).

### 3.5. Liver Gene Expression of *T. ovatus*

**Figure 3:**
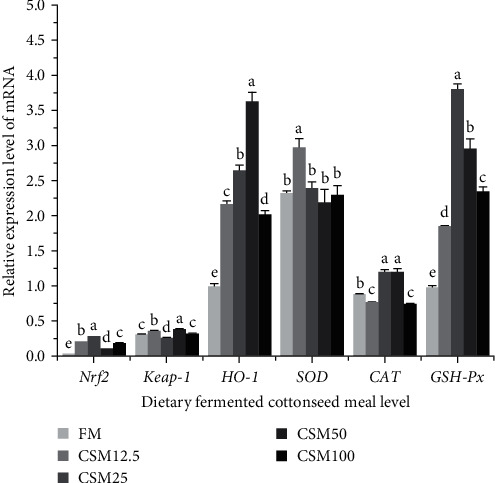
Effect of FCSM substituting fish meal on hepatic antioxidant-related gene expression. Data are expressed as means ± SEM (*n* = 3). Means with different superscripts are significantly different (*P* < 0.05).

As shown in Figure 3, the relative gene expression of *Nrf2* of fish fed with CSM25 diet was higher than that of the other groups (*P* < 0.05). The relative gene expression of *Keap-1* of fish fed with CSM25 diet was lower than those in FM (*P* < 0.05). The relative gene expression of *HO-1* of fish fed with CSM50 diet was higher than that of the other groups. The relative gene expression of *SOD* of fish fed with CSM12.5 diet was significantly higher than those in other groups (*P* < 0.05). The relative gene expression of *CAT* of fish fed with CSM25 and CSM 50 diets was significantly higher than those in other groups (*P* < 0.05). The relative gene expression of *GSH-Px* of fish fed with CSM25 diet was significantly higher than those in other groups (*P* < 0.05).

**Figure 4:**
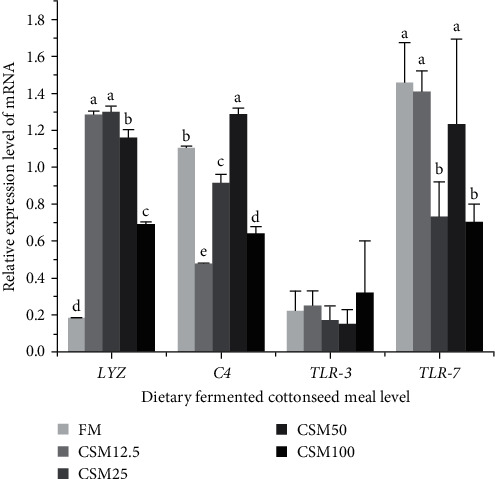
Effect of FCSM substituting fish meal on liver-related gene expression. Data are expressed as means ± SEM (*n* = 3). Means with different superscripts are significantly different (*P* < 0.05).

As shown in Figure 4, the relative gene expression of *LYZ* of fish fed with CSM12.5 and CSM 25 diets was higher than that of the other groups. The relative gene expression of *C4* of fish fed the CSM12.5 diet was significantly lower than those in other groups (*P* < 0.05). No significant difference was observed in the relative expression of *TLR-3* among all treatments (*P* > 0.05). The relative gene expression of *TLR-7* of fish fed with CSM25 and CSM100 diets was significantly lower than those in other groups (*P* < 0.05).

### 3.6. Muscle Gene Expression of *T. ovatus*

**Figure 5:**
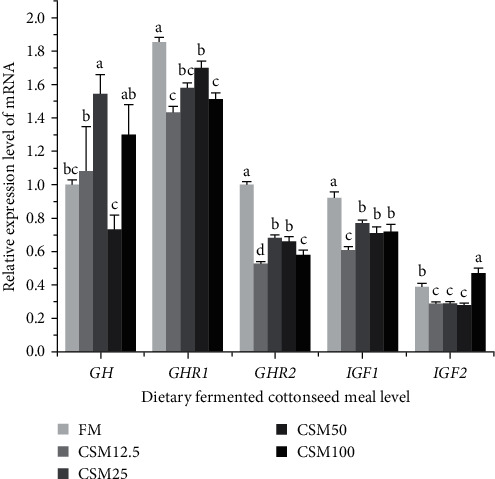
Effect of FCSM substituting fish meal on the expression of muscle growth-related genes. Data are expressed as means ± SEM (*n* = 3). Means with different superscripts are significantly different (*P* < 0.05).

As shown in Figure 5, the relative gene expression of *GH* of fish fed with CSM25 diet was significantly higher than those in FM, CSM12.5, and CSM50 (*P* < 0.05). The relative gene expression of *GHR1* of fish fed with FM diet was significantly higher than those in other groups (*P* < 0.05). The relative gene expression of *GHR2* and *IGF1* of fish fed with FM diet was significantly higher than those in other groups (*P* < 0.05). The relative gene expression of *IGF2* of fish fed with CSM12.5, CSM25, and CSM50 diets was significantly lower than those in other groups (*P* < 0.05).

**Figure 6:**
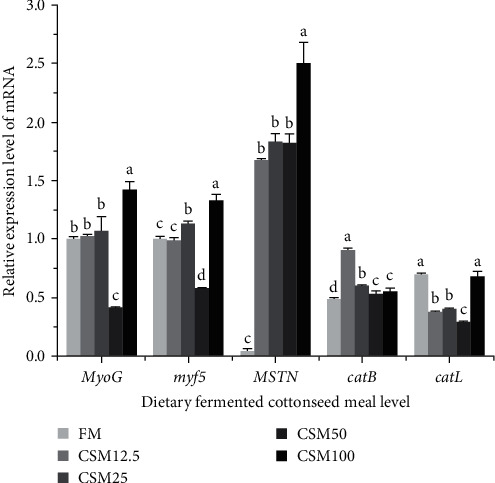
Effect of FCSM substituting fish meal on the expression of genes related to muscle quality. Data are expressed as means ± SEM (*n* = 3). Means with different superscripts are significantly different (*P* < 0.05).

As shown in Figure 6, the relative gene expression of *MyoG*, *myf5*, and *catL* of fish fed with CSM50 diet was significantly lower than those in other groups (*P* < 0.05). The relative gene expression of *MSTN* of fish fed with CSM100 diet was significantly higher than those in other groups (*P* < 0.05). The relative gene expression of *catB* of fish fed with FM diet was significantly lower than those in the other groups (*P* < 0.05).

## 4. Discussion

The findings of this experiment showed that when the replacement of FM with FCSM reached 100%, the SR decreased to 74.7%. In addition, both WGR and SGR were lower compared to the other groups. Moreover, the growth of golden pompano was negatively affected. As the percentage of replacement of FM with FCSM ranged from 12.5% to 50%, no significant changes were observed in SR, FI, FCR, PER, HSI, and VSI of golden pompano compared to FM. This result suggests that the inclusion of plant protein in the diet of golden pompano can effectively reduce the use of fishmeal while improving the growth of golden pompano to some extent. When the replacement of FM with FCSM reached 100%, the SR, WGR, SGR, and PER decreased. This could be because the amount of free cottonseed phenol in the diet gradually increased as the replacement of FM with FCSM in the diet increased, and the excessive intake of free cottonseed phenol by golden pompano caused the fish to grow slowly. The result indicated that replacing more than 20% of fishmeal with low phenol cottonseed meal had a significant effect on the intestinal barrier function of juvenile golden pompano and caused an imbalance in the intestinal microbiota [33]. Alterations in intestinal health conditions will have a significant impact on both animal growth performance and immunological function [34, 35]. However, substitution of a high proportion of FM with FCSM negatively affects the intestinal microflora and nutrient absorption capacity of fish [36]. The observed decline in growth performance in golden pompano may be attributed to this intestinal damage. A quadratic regression analysis on WGR and SGR indicated that the optimal dietary FM replacement by FCSM, for the optimal growth of juvenile golden pompano was 29.38% and 24.74%, respectively.

Carotenoids are important nutrients that affect the color change in fish [37], but fish cannot synthesize carotenoids by themselves, and cottonseed meal contains high levels of carotenoids [38]. In this experiment, the *a* ^*∗*^ of the dorsal skin of fish fed the CSM100 diet was significantly higher than those in CSM12.5. At present, there are few studies on the effect of FCSM on abdominal and dorsal skin changes in aquatic animals. The results of a previous study investigating the replacement of soybean meal by cottonseed meal in the diet of *Ictalurus punctatus* are consistent with the present investigation [39]. The presence of carotenoids in cottonseed meal may be responsible for the changes in the pigmentation of the abdominal and dorsal skin of the golden pompano. However, more research is needed to thoroughly investigate this phenomenon.

Typically, the levels of ALT and ALP in the blood are modest. However, when liver cells become inflamed or injured, a significant amount of ALT is released into the bloodstream, resulting in an elevated concentration of aminotransferases in the blood [40]. In the present study, the ALT of fish fed the CSM100 diet was significantly higher than those in FM. These results indicate that replacement of fishmeal with FCSM resulted in increased levels of blood ALT, leading to liver damage in golden pompano. The activity of ALP is associated with the nutritional and immunological status of the body [41], and it functions as a detoxifying enzyme in the body. Under typical circumstances, the activity of blood ALP activity is minimal. However, when there are liver or bone abnormalities, the activity of serum ALP increases [42]. In the present study, ALP of fish fed with CSM12.5 and CSM100 diets was significantly higher than those in FM. GLU in serum is an essential source of energy for all tissues and organs in the body, so the dynamic balance of glucose content plays a vital role in maintaining the normal physiological functions of the body [43]. GLU is released from hepatocytes into serum, resulting in increased glutathione activity in the serum [44]. In this experiment, the concentration of GLU in serum increased with the increase in the proportion of fishmeal replaced by FCSM, which was different from the results of golden pompano by Shen et al. [42]. The decrease in free cotton phenol content after cottonseed meal fermentation may result in a lesser effect on glucose metabolism in fish. TC is the primary expression of nutrients in the blood after being absorbed by the fish body during lipid metabolism [45], and its concentration is one of the essential indicators to determine the function of lipid metabolism in the body. When the replacement ratio of FM with FCSM was less than 50%, the concentration of TG in the serum of golden pompano was significantly lower than those in FM, and only that of the CSM100 group was significantly higher than those in FM. On the other hand, FCSM is a plant protein, which may stimulate the increase of adrenaline secretion with the increase of substitution concentration, leading to the increase of blood glucose. The level of TC concentration could reflect the lipid absorption status [46]. In this study, TC concentrations were significantly lower in the CSM25 and CSM50 groups and significantly higher in the CSM12.5 and CSM100 groups than in the FM group. This result suggests that the total replacement of FM with FCSM leads to impaired liver function in golden pompano. The decrease in TC content in serum could be because the replacement of FM with FCSM affected the digestion and absorption of the intestine of golden pompano, or it could be that the cholesterol content in FCSM was less, which led to the decrease in cholesterol content in the diet after the replacement of FM [47]. Cholesterol is mainly absorbed in the upper part of the small intestine [48]. Serum high-density lipoprotein cholesterol (HDL) and low-density lipoprotein cholesterol (LDL) have opposite functions, with the former transporting cholesterol from the body back to the liver for metabolism and the latter transporting endogenous cholesterol synthesized by the liver to the tissues [49]. In the present study, HDL concentrations were significantly higher in the CSM12.5 and CSM25 groups than that in the FM group. LDL of fish fed the CSM12.5, CSM25, and CSM50 diets were significantly higher than those in FM, suggesting that the replacement of FM with FCSM may have increased the rate at which golden pompano lipids are transported back to the liver for metabolism. Complement is a protein with enzymatic activity that assists and complements specific antibodies and plays a role in ammonolysis and hemolysis [50]. In the present study, the levels of complement 3 in the CSM25, CSM50, and CSM100 groups were not significantly different from those in the FM group, and the levels of complement 4 in the CSM12.5, CSM25, and CSM100 groups. Some researchers suggested that increased glutamic aminotransferase and ALP activities and increased complement 3 and 4 concentrations could reduce bacterial and viral infections in grass carp [51]. A study conducted on juvenile common carp (*Cyprinus carpio*) revealed that consuming excessive amounts of cottonseed meal in their diet may result in mild liver damage [52]. Therefore, we found that excessive replacement of FM with FCSM resulted in liver damage in golden pompano.

There are many types of enzymes in the antioxidant system, among which SOD is the first to be activated because it can convert superoxide anions in the organism to hydrogen peroxide for removal [53]. The primary function of peroxidase is to catalyze the decomposition of H_2_O_2_ into H_2_O and O_2_ and to prevent the conversion of H_2_O_2_ into OH ions. Therefore, it is one of the necessary antioxidant enzymes in the antioxidant system of fish [54]. This experiment indicated that no significant difference was observed in SOD and CAT among all treatments. The primary function of GSH-Px is to protect the structure and function of cell membranes by eliminating hydrogen peroxide and lipid peroxidation products produced during cellular respiratory metabolism through the catalysis of reduced glutathione, thereby reducing the peroxidation of polyunsaturated fatty acids in cell membranes [55]. The level of T-AOC reflects the ability of the organism to scavenge reactive oxygen species [56]. This result indicated that T-AOC of fish fed the CSM25 diet was significantly higher than those in CSM50. In typical organisms, a dynamic balance between the production and elimination of reactive oxygen species should be maintained, and excessive amounts of reactive oxygen species inevitably cause cellular damage [57]. This result indicated that GSH-Px of fish fed the CSM50 diet was significantly lower than those in FM. MDA is a product of lipid peroxidation, and when the organism is threatened by endogenous oxidative stress, MDA will accumulate in the cells, and its level can indirectly determine the degree of oxygen radical damage suffered by the cells of the organism [53]. This result indicated that no significant difference in MDA was observed among all treatments. The highest MDA levels were observed in fish fed the CSM50 and the CSM100 diets. These results could be explained by the fact that high levels of dietary CSM may reduce the antioxidant capacity of juvenile golden pompano, which was similar to the results of a research that examined the replacement of FM with cottonseed meal in the diet of juvenile ussuri catfish (*Pseudobagrus ussuriensis*) [58].

The antioxidant response in fish is highly complex and involves the activation of multiple signaling pathways when oxidative stress disrupts the balance between intracellular oxidative and antioxidant systems. This regulation primarily involves changes in the activities of existing antioxidant enzymes and the synthesis of new molecules [59]. Previous research has shown that the *Nrf2-ARE* signaling pathway activates the transcription of antioxidant enzyme genes, acting as an intermediate in the defense against oxidative stress [60]. Keap1 is a protein that interacts with *Nrf2* in the cytoplasm under normal physiological conditions [61]. Upon stimulation, the interaction between *Nrf2* and *Keap1* is disrupted. The activated *Nrf2* then translocates to the nucleus and binds to antioxidant response elements, thereby regulating the transcription of antioxidant-related genes [62]. In the present study, substitution of FM with FCSM up to 25% caused an increase in the relative expression of *Nrf2* and a decrease in the relative expression of *Keap1* compared with the control group, indicating that substitution FM with FCSM up to 25% activates the *Nrf2/Keap1* pathway. Similar results were observed in a study on the replacement of fishmeal with cottonseed protein concentrate in the diet of juvenile *Songpu mirror carp* [63]. In heme catabolism, heme oxygenase 1 (*HO-1*) is the rate-limiting enzyme, and *HO-1* expression is regulated by various intracellular signaling molecules [64]. Increased *HO-1* gene expression and its resulting enzyme activity can protect against cell or tissue damage caused by oxidative stress [65]. The *Nrf2/HO-1* signaling pathway has been shown to reduce oxidative stress and inflammation in the liver, thus contributing significantly to the maintenance of stable liver function [66]. In the present study, substitution of FM with FCSM up to 25% and 50% caused an increase in the relative expression of *HO-1* mRNA compared with the control group, indicating that the appropriate ratio of FM to FCSM attenuated liver inflammation. However, substitution of FM with FCSM up to 100% caused a decrease in the relative expression of *HO-1* compared with the CSM25 and CSM50 groups, indicating that the oversubstitution of FCSM caused liver damage. Similar results were observed in the studies on the replacement of fish meal with squid peptide in the diets of *Litopenaeus vannamei* [67].

Activation of the *Nrf2–Keap1* signaling pathway stimulates the production of antioxidant genes such as *SOD*, *CAT*, and *GSH-Px* [68]. *SOD* is an essential enzyme that plays a key role in the body's initial defense mechanism against the generation of free radicals and other reactive substances within cells [65]. In the present study, the expression of *SOD* was significantly higher in the CSM12.5 group than those in the other groups, indicating that the replacement of modest amounts of FM with FCSM enhances the antioxidant capacity of the liver. In a study on the replacement of fishmeal with pork meal in golden pompano, liver *SOD* expression was slightly increased in the 10% replacement ratio group [62]. *CAT* and *GSH-Px* decompose H_2_O_2_ and protect tissues from highly reactive hydroxyl radicals [69]. In this study, the expression of *CAT* and *GSH-Px* was significantly higher in the CSM25 group than those in the other groups. This indicates that the use of an appropriate amount of FCSM can enhance the expression of *CAT* and *GSH-Px*, leading to improved antioxidant capacity in the liver. Similar results were observed in the studies on golden pompano, where the expression of *CAT* and *GSH-P*x of the replacement group was higher than that of all other groups [33]. Gene expression and associated enzyme activities are inconsistent, this may be the result of enzyme activity modulation at the posttranslational level [70]. It could also be due to the fact that the mRNA level of an antioxidant enzyme represents only a snapshot of activity at a given time, while there is a time lag between transcription and translation [71]. The waterborne column environment may also contribute to inconsistent gene expression and associated enzyme activities [72]. In the present study, we found inconsistencies in *SOD*, *CAT*, and *GSH-Px* activities and gene expression levels. Similar results have been reported in *Anguilla Anguilla* [73] and *Pseudosciaena Crocea* [74]. The reasons for this need to be verified in further experiments.

*LZM* has bacteriolytic properties and serves as a critical enzyme in the immune defense mechanism of fish against bacterial infections. The level of *LZM* activity is considered an important indicator of fish immunity [75]. In this experiment, the relative gene expression of *LYZ* of fish fed with CSM12.5 and CSM 25 diets was higher than that of the other groups. *C4* is a globulin found in fish that exhibits zymogenic activity, with the purpose of eliminating or killing harmful bacteria [76]. In this experiment, the relative gene expression of *C4* of fish fed the CSM12.5 diet was significantly lower than those in other groups. PAMPs are recognized by *TLRs* and form the first line of defense against infection [77]. *TLRs* have the ability to recognize PAMPs, activate MyD88, and send signals to nucleus to initiate an inflammatory response [78]. In this experiment, the relative gene expression of *TLR-7* of fish fed with CSM25 and CSM100 diets was significantly lower than those in other groups, no significant difference was observed in the relative expression of *TLR-3* among all treatments. This suggests that the replacement of FM with FCSM may lead to inflammation in the liver of golden pompano. The previous research found that feeding *S. sihama* with increased amounts of low-gossypol cottonseed meal had adverse effects on the organism's growth and impaired the structural integrity of the liver [79].

The growth hormone/insulin-like growth factor (GH/IGFs) axis in fish can regulate their growth and development [80]. In combination with *GHR*, *GH* may be involved in several physiological regulatory effects, including growth, immunity, and metabolism [81]. Tissue expression profiles of *GHR* genes have also been identified in various Scleractinia fishes, and *GHR*s are widely expressed in several tissues [82]. In this experiment, the relative gene expression of *GH* of fish fed with CSM50 diet was significantly lower than those in other groups, the relative gene expression of *GHR1* of fish fed with FM diet was significantly higher than those in other groups. This indicates that the replacement of fishmeal with FCSM within an appropriate range can promote growth. The *IGFs* system plays a vital role in the regulation of vertebrate growth and development [83], and the insulin-like growth factors in this system include *IGF1*, *IGF2*, and *IGF3*. *IGF1* is thought to be extensively involved in growth regulation at all stages of vertebrate growth [84], and *IGF2* is believed to be mainly associated with the early stages of growth and development of the species [85], while *IGF3* is thought to play an important role mainly in ovarian function [86]. In the present study, the relative gene expression of *GHR2* and *IGF1* of fish fed with FM diet was significantly higher than those in other groups, the relative gene expression of *IGF2* of fish fed with CSM12.5, CSM25, and CSM50 diets was significantly lower than those in other groups. This result indicated that substituting FCSM for FM had no significant effect on the expression of *GH* genes but significantly downregulated the expression of *GHR1*, *GHR2*, *IGF1*, and *IGF2* genes.

Myostatin (*MSTN*) genes play a critical role in the regulation of muscle processes [87]. Myogenic factor 5 (*Myf5*) regulates myocyte regeneration and dynamic homeostasis [88]. Myogenin (*MyoG*) is responsible for muscle differentiation [89], while MSTN negatively regulates skeletal muscle growth and development by preventing myocyte and muscle fiber growth. Cathepsin B (*CatB*) and Cathepsin L (*CatL*) are important members of the cysteine protease family that are mainly found in lysosomes and are involved in a variety of physiological responses. When released from lysosomes, they can disrupt muscle protein integrity and accelerate muscle softening [90]. The relative gene expression of *MyoG*, *myf5*, and *catL* of fish fed the CSM50 diet was significantly lower than those in other groups. The relative gene expression of *MSTN* of fish fed with CSM100 diet was significantly higher than those in other groups. The relative gene expression of *catB* of fish fed with FM diet was significantly lower than those in other groups. This may be because, with the increase of FCSM substitution ratio in the diet, the excessive intake of FCSM in golden pompano leads to a change in muscle quality. The result indicated that replacing FM with cottonseed meal in the diet of juvenile crayfish *Procambarus clarkii* affected growth performance, feed utilization, and muscle parameters [25]. The research indicated that including a significant amount of cottonseed meal (27%) in the diet had a negative effect on growth performance and muscle texture of *grass carp* [26]. These results are similar to the present experiment.

## 5. Conclusion

Replacing 12.5%–50% FM with FCSM as the dietary protein source did not significantly affect the SR, WGR, SGR, VSI, HSI, CF, FI, FCR, and PER of juvenile golden pompano. At 100% replacement, biochemical indices, liver antioxidant capacity, and muscle quality of juvenile golden pompano were negatively affected. Quadratic regression analysis on WGR and SGR indicated that the optimum levels of dietary FM substitution by FCSM for optimal growth of juvenile golden pompano were 29.38% and 24.74%, respectively. Based on the results of this study, it is recommended that 24.74%–29.38% of FM should be replaced by FCSM in the diet of golden pompano.

## Figures and Tables

**Table 1:** Formulation and nutrients levels of the experimental diets (percentage of dry matter).

Item	Fermented cotton meal replacement percentage (%)				
	FM	CSM12.5	CSM25	CSM50	CSM100
Ingredients					
Fish meal	400	350	300	200	0
Soy protein concentrate	160	160	160	160	160
Soybean meal	40	40	40	40	40
Fermented cotton meal	0	51	102	205	409
Corn starch	177	162	147	117	58
Porcine blood cell protein powder	20	20	20	20	20
Beer yeast powder	20	20	20	20	20
Fish oil	73	77	81	89	105
Vitamin and mineral premix^1^	10	10	10	10	10
Ca(H_2_PO_4_)_2_	5	5	5	5	5
Choline chloride	5	5	5	5	5
Lecithin	10	10	10	10	10
Microcrystalline cellulose	75	84	91	107	138
Betaine	5	5	5	5	5
Lysine	0	1	3	5	10
Methionine	0	0	1	2	5
Total	1,000	1,000	1,000	1,000	1,000
Nutrient levels					
Ash	12.08	12.03	12.01	10.35	7.99
Crude protein	43.89	43.65	43.31	43.62	43.49
Crude lipid	11.30	11.54	11.71	11.85	12.02
Crude fiber	7.29	7.83	8.46	10.04	12.93
Nitrogen free extract	24.71	24.95	24.52	24.13	23.57
Free gossypol (mg/kg)	64.7	95.9	88.6	171.0	190.0

^1^Vitamin and mineral premix provided by Shenzhen Jingji Zhinong Times Co., Ltd., (mg/kg diet): The formulation includes the following amounts of vitamins and minerals per kilogram: Vitamin A at a minimum of 450,000 IU, Vitamin B1 at a minimum of 1,000 mg, Vitamin B2 at a minimum of 1,000 mg, Vitamin B6 at a minimum of 1,500 mg, Vitamin B12 at a minimum of 5 mg, Vitamin K3 at a minimum of 800 mg, inositol at a minimum of 12,000 mg, D-pantothenic acid at a minimum of 3,500 mg, nicotinic acid at a minimum of 2,000 mg, folic acid at a minimum of 500 mg, D-biotin at a minimum of 5 mg, Vitamin D3 at a range of 300,000–400,000 IU, Vitamin E at a minimum of 8,000 IU, Na_2_SeO_3_ at 20 mg, CuSO_4_ · 5H_2_O at 24 mg, FeSO_4_ · H_2_O at 266.65 mg, ZnSO_4_ · H_2_O at 100 mg, MnSO_4_ · H_2_O at 120 mg, Ca (IO_3_)_2_ at 50 mg, CoSO_4_·7H_2_O at 10 mg, Mg at 20 g, and zeolite at 4,380.55 mg.

**Table 2:** Amino acid compositions (g/100 g) of experimental diets (dry matter basis).

AA/∑AA	Fermented cotton meal replacement percentage (%)				
	FM	CSM12.5	CSM25	CSM50	CSM100
EAA					
Lysine	2.02	1.98	1.98	1.89	1.59
Arginine	1.69	1.77	1.90	2.19	2.60
Methionine	0.54	0.51	0.57	0.52	0.59
Threonine	1.15	1.14	1.13	1.08	0.95
Isoleucine	1.09	1.08	1.04	0.98	0.87
Leucine	2.17	2.17	2.13	2.09	1.88
Phenylalanine	1.13	1.19	1.23	1.30	1.37
Valine	1.42	1.41	1.38	1.34	1.26
Histidine	0.72	0.74	0.75	0.76	0.74
∑EAA	11.93	11.99	12.11	12.15	11.85
NEAA					
Aspartic acid	2.73	2.71	2.72	2.72	2.54
Serine	1.04	1.07	1.11	1.15	1.10
Glutamic acid	3.92	4.07	4.26	4.47	4.30
Glycine	1.63	1.49	1.44	1.25	0.83
Alanine	1.72	1.72	1.67	1.54	1.28
Cystine	0.24	0.25	0.26	0.26	0.26
Tyrosine	0.69	0.68	0.72	0.71	0.72
Proline	1.44	1.41	1.35	1.30	1.18
∑NEAA	13.41	13.4	13.53	13.4	12.21
∑AA	25.3	25.4	25.6	25.6	24.1

**Table 3:** The primers for real-time fluorescence quantification PCR.

Gene	Sequence	Reference
GH-qF	GCCAGTCAGGACGGAG	[25]
GH-qR	AGGAGGCGGGGCTACA	

GHR1-qF	GGTGGAGTTCATTGAGGTGGAT	[25]
GHR1-qR	TGGTGGCTGACAGGTTGG	

GHR2-qF	CACCACCTCTACCTCCTCTG	[25]
GHR2-qR	CCCTCTTCGGCGTTCATA	

IGF1-qF	GACGCTTACAGGAGGAGAA	[25]
IGF1-qR	GCTGCTGGATGTGTTCAC	

IGF2-qF	CTGTGACCTCAACCTGCT	[25]
IGF2-qR	CTCTGCCACTCCTCGTATT	

*β*-actin-qF	TACGAGCTGCCTGACGGACA	[26]
*β*-actin-qR	GGCTGTGATCTCCTTCTGCA	

myoG-qF	AACCAGAGGCTGCCCAAGG	[27]
myoG-qR	GCTGTCCCGTCTCAGTGTCC	

myf5-qF	AAGAACGAGAGTTTGGGCGA	[27]
myf5-qR	AGGACGTGGTATATGGGCCT	

MSTN-qF	GACGGGAACAGGCACATACG	[27]
MSTN-qR	GCAGCCACACGGTCAACACT	

CatL-qF	CCACTGGCACCTCTGCAAGA	[27]
CatL-qR	GCCCGTAGCACTGTTTGCCC	

catB-qF	TCTGCCTGGGACTTCTGGACCA	[27]
catB-qR	ACACTTGAGGACGCACTGAG	

lyz-qF	GGAGTCTGGTGTTTCTGCTCTTTG	Self-designed based on transcripts
lyz-qR	GGTGGCTCTAGTGTTGTAGTTCG	

C4-qF	TGGAGAAAAAGTTAAAGGGGC	[28]
C4-qR	CAGGAAGGAAGTATGAGCGAGT	

TLR3-qF	TCTCCTCAACTTTCTCCACG	Self-designed based on transcripts
TLR3-qR	GAACCACATTATGCTCTCGC	

TLR7-qF	ACCTGCTCTACTCCACTG	Self-designed based on transcripts
TLR7-qR	CCTGAACTCTAACCTCTC	

Nrf2-qF	TTGCCTGGACACAACTGCTGTTAC	[29]
Nrf2-qR	TCTGTGACGGTGGCAGTGGAC	

Keap1-qF	CAGATAGACAGCGTGGTGAAGGC	[29]
Keap1-qR	GACAGTGAGACAGGTTGAAGAACTCC	

HO-1-qF	AGAAGATTCAGACAGCAGCAGAACAG	[29]
HO-1-qR	TCATACAGCGAGCACAGGAGGAG	

SOD-qF	CCTCATCCCCCTGCTTGGTA	[30]
SOD-qR	CCAGGGAGGGATGAGAGGTG	

GSH-Px-qF	GCTGAGAGGCTGGTGCAAGTG	[31]
GSH-Px-qR	TTCAAGCGTTACAGCAGGAGGTTC	

CAT-qF	GGATGGACAGCCTTCAAGTTCTCG	[30]
CAT-qR	TGGACCGTTACAACAGTGCAGATG	

GH, growth hormone gene; GHR1, growth hormone receptor 1 gene; GHR2, growth hormone receptor 2 gene; IGF1, insulin-like growth factor 1 gene; IGF2, insulin-like growth factor 2 gene; MyoG, myogenin gene; myf5, myogenic factor 5 gene; MSTN, myostatin gene; catL, cathepsin L gene; catB, cathepsin B gene; LYZ, lysozyme gene; C4, C4 complement gene; TLR-3, toll-like receptor 3 gene; TLR-7, toll-like receptor 7 gene; Nrf2, nuclear factor erythroid 2-related factor 2 gene; Keap1, Kelch-like ECH-associated protein 1 gene; HO-1, heme oxygenase 1 gene; SOD, superoxide dismutase gene; GSH-Px, glutathione peroxidase gene; and CAT, catalase gene.

**Table 4:** Effects of FCSM substituting fish meal on growth performance of *T. ovatus*.

Item	Fermented cotton meal replacement percentage (%)				
	FM	CSM12.5	CSM25	CSM50	CSM100
SR (%)	89.33 ± 5.33^a^	94.67 ± 1.33^a^	98.67 ± 1.33^a^	96 ± 2.31^a^	74.67 ± 6.67^b^
WGR (%)	621 ± 26^b^	671 ± 6^ab^	698 ± 16^a^	642 ± 33^ab^	389 ± 13^c^
SGR (%)	3.52 ± 0.06^b^	3.65 ± 0.02^ab^	3.71 ± 0.04^a^	3.58 ± 0.08^ab^	2.83 ± 0.05^c^
VSI (%)	7.63 ± 0.57^b^	7.63 ± 0.75^b^	7.67 ± 0.21^b^	8.00 ± 0.76^ab^	8.83 ± 0.45^a^
HSI (%)	1.43 ± 0.15^b^	1.47 ± 0.15^b^	1.27 ± 0.12^b^	1.40 ± 0.17^b^	2.77 ± 0.65^a^
CF (%)	3.67 ± 0.06^b^	3.67 ± 0.21^b^	4.30 ± 0.35^a^	3.90 ± 0.26^ab^	3.63 ± 0.12^b^
Per fish FI (g)	21.77 ± 0.42^a^	22.17 ± 0.39^a^	22.50 ± 0.38^a^	21.90 ± 0.51^a^	19.27 ± 1.33^b^
FCR (%)	1.33 ± 0.04^b^	1.25 ± 0.01^b^	1.22 ± 0.01^b^	1.29 ± 0.04^b^	1.89 ± 0.16^a^
PER (%)	1.81 ± 0.05^a^	1.93 ± 0.03^a^	1.98 ± 0.02^a^	1.88 ± 0.07^a^	1.29 ± 0.11^b^

Data are expressed as means ± SEM (*n* = 3). Means with different superscripts are significantly different (*P* < 0.05). SR, survival rate; WGR, weight gain rate; SGR, specific growth rate; VSI, viscerosomatic index; HSI, hepatosomatic index; CF, condition factor; FI, feed intake; FCR, feed conversion ratio; and PER, protein efficiency ratio.

**Table 5:** Effects of FCSM substituting fish meal on body coloration of *T. ovatus*.

Item	Fermented cotton meal replacement percentage (%)				
	FM	CSM12.5	CSM25	CSM50	CSM100
Dorsal skin					
*L* ^*∗*^	65.51 ± 4.44	65.94 ± 4.92	60.94 ± 3.81	63.15 ± 4.51	61.7 ± 3.43
*a* ^*∗*^	−1.91 ± 1.39^ab^	−2.1 ± 1.13^b^	−1.08 ± 0.66^ab^	−1.55 ± 0.78^ab^	−0.73 ± 0.74^a^
*b* ^*∗*^	9.18 ± 2.03	8.74 ± 1.68	9.7 ± 2.97	9.86 ± 2.18	10.69 ± 1.87
Abdominal skin					
*L* ^*∗*^	80.4 ± 3.07	74.96 ± 25.15	78.54 ± 2.63	81.1 ± 2.38	79.06 ± 2.12
*a* ^*∗*^	−0.46 ± 0.62	−0.68 ± 0.45	−0.48 ± 0.25	−0.57 ± 0.56	−1.04 ± 0.56
*b* ^*∗*^	7 ± 1.86	6.83 ± 1.29	6.85 ± 1.77	6.64 ± 1.30	7.18 ± 2.32

*L* ^*∗*^, brightness; *a* ^*∗*^, redness; and *b* ^*∗*^, yellowness. Data are expressed as means ± SEM (*n* = 3). Means with different superscripts are significantly different (*P* < 0.05).

**Table 6:** Effects of FCSM substituting fish meal on blood physiological indices of *T. ovatus*.

Enzyme	Fermented cotton meal replacement percentage (%)				
	FM	CSM12.5	CSM25	CSM50	CSM100
ALT (U/L)	3.92 ± 0.5^c^	3.85 ± 0.24^c^	5.65 ± 0.32^b^	5.16 ± 0.49^b^	7.35 ± 0.42^a^
ALP (U/L)	21.61 ± 0.83^d^	37.46 ± 0.72^a^	29.28 ± 0.16^c^	33.13 ± 0.21^b^	36.14 ± 0.47^a^
GLU (mmol/L)	3.795 ± 0.003^e^	5.643 ± 0.005^d^	7.085 ± 0.084^c^	8.368 ± 0.022^b^	8.705 ± 0.083^a^
TG (mmol/L)	3.898 ± 0.017^b^	1.940 ± 0.006^e^	3.838 ± 0.024^c^	3.555 ± 0.009^d^	4.258 ± 0.014^a^
TC (mmol/L)	2.938 ± 0.013^c^	3.048 ± 0.023^b^	2.800 ± 0.018^d^	2.540 ± 0.027^e^	3.565 ± 0.025^a^
HDL (mmol/L)	1.160 ± 0.004^c^	1.458 ± 0.002^a^	1.235 ± 0.006^b^	1.105 ± 0.009^d^	1.170 ± 0.026^c^
LDL (mmol/L)	0.765 ± 0.006^a^	0.430 ± 0.007^d^	0.670 ± 0.012^b^	0.543 ± 0.008^c^	0.775 ± 0.012^a^
C3 (g/L)	0.0461 ± 0.0038^a^	0.0362 ± 0.0011^b^	0.0486 ± 0.0002^a^	0.0452 ± 0.002^a^	0.0426 ± 0.0034^ab^
C4 (g/L)	0.0190 ± 0.0011^a^	0.0167 ± 0.0000^bc^	0.0166 ± 0.0005^bc^	0.0179 ± 0.0004^ab^	0.0152 ± 0.0006^c^

Data are expressed as means ± SEM (*n* = 3). Means with different superscripts are significantly different (*P* < 0.05). ALT, alanine aminotransferase; ALP, alkaline phosphatase; GLU, glucose; TG, triglyceride; TC, total cholesterol; HDL, high-density lipoprotein; LDL, low-density lipoprotein; C3, complement 3; and C4, complement 4.

**Table 7:** Effects of FCSM substituting fish meal on hepatic antioxidant indices of *T. ovatus*.

Enzyme	Fermented cotton meal replacement percentage (%)				
	FM	CSM12.5	CSM25	CSM50	CSM100
SOD (U/mg)	5.51 ± 0.56	5.72 ± 1.11	4.91 ± 0.36	4.04 ± 1.32	3.9 ± 0.88
CAT (U/mg)	2.01 ± 0.13	1.84 ± 0.58	2.05 ± 0.35	1.85 ± 0.14	1.79 ± 0.08
GSH-Px (U/mg)	18.84 ± 4.09^a^	12.97 ± 6.05^ab^	11.87 ± 2.34^ab^	9.33 ± 1.76^b^	13.47 ± 0.76^ab^
T-AOC (U/mg)	0.59 ± 0.04^ab^	0.48 ± 0.16^ab^	0.63 ± 0.06^a^	0.34 ± 0.05^b^	0.43 ± 0.13^ab^
MDA (nmol/mg)	0.25 ± 0.07	0.25 ± 0.10	0.33 ± 0.04	0.34 ± 0.04	0.34 ± 0.07

Data are expressed as means ± SEM (*n* = 3). Means with different superscripts are significantly different (*P* < 0.05). SOD, superoxide dismutase; CAT, catalase; GSH-Px, glutathione peroxidase; T-AOC, total antioxidant capacity; and MDA, malondialdehyde.

## Data Availability

Data for this research article are available from the corresponding author upon reasonable request.
